# Antioxidant, Organoleptic and Physicochemical Changes in Different Marinated Oven-Grilled Chicken Breast Meat

**DOI:** 10.3390/foods11243951

**Published:** 2022-12-07

**Authors:** Charles Odilichukwu R. Okpala, Szymon Juchniewicz, Katarzyna Leicht, Małgorzata Korzeniowska, Raquel P. F. Guiné

**Affiliations:** 1 Department of Functional Food Products Development, Faculty of Biotechnology and Food Science, Wrocław University of Environmental and Life Sciences, 51-630 Wrocław, Poland; 2 UGA Cooperative Extension, College of Agricultural and Environmental Sciences, University of Georgia, Athens, GA 30602, USA; 3 CERNAS—Research Centre for Natural Resources, Environment and Society, Polytechnic Institute of Viseu, 3504-510 Viseu, Portugal

**Keywords:** oven-grill, herbs, spices, meat processing, product development

## Abstract

The antioxidant, organoleptic, and physicochemical changes in different marinated oven-grilled chicken breast meat were investigated. Specifically, the chicken breast meat samples were procured from a local retailer in Wroclaw, Poland. The antioxidant aspects involved 2,2′-azinobis-(3-ethylbenzthiazolin-6-sulfonic acid) (ABTS), 1,1-diphenyl-2-pierylhydrazy (DPPH), and ferric-reducing antioxidant power (FRAP). The organoleptic aspects involved sensory and texture aspects. The physicochemical aspects involved the pH, thiobarbituric acid reactive substance (TBARS), cooking weight loss, L* a* b* color, and textural cutting force. Different marination variants comprised incremental 0.5, 1, and 1.5% concentrations of Baikal skullcap (BS), cranberry pomace (CP), and grape pomace (GP) that depicted antioxidants, and subsequently incorporated either African spice (AS) or an industrial marinade/pickle (IM). The oven grill facility was set at a temperature of 180 °C and a constant cooking time of 5 min. Results showed various antioxidant, organoleptic and physicochemical range values across the different marinated oven-grilled chicken breast meat samples, most of which appeared somewhat limited. Incorporating either AS or IM seemingly widens the ABTS and FRAP ranges, with much less for the DPPH. Moreover, with increasing CP, GP, and BS concentrations, fluctuations seemingly persist in pH, TBARS, cooking weight loss, L* a* b* color, and textural cutting force values even when either AS or IM was incorporated, despite resemblances in some organoleptic sensory and texture profiles. Overall, the oven-grilling approach promises to moderate the antioxidant, organoleptic, and physicochemical value ranges in the different marinated chicken breast meat samples in this study.

## 1. Introduction

Within the European Union, particularly Poland, chicken meat thrives industrially through poultry production, which as of 2021 had amounted to an excess of 2.9 million metric tons [1,2]. Besides the poultry production system in Poland delivering a promising product quality [3], factors that influence the chick quality would include the assessment of day-olds, broiler breeder nutrition, flock age, egg storage, and its incubation/post-hatching period(s), as well as in ovo-feeding [4]. The passion for healthy living across the globe is among the keys that drive the steady increase in poultry meat production. Additionally, factors influencing the consumption of poultry meat products include visual and smell impressions, the color of the meat/carcass, the conditions of birds’ housing/production systems, as well as the quality of the product/raw material contribute to the increasing poultry meat consumption [2]. Both the influencing factors of chick quality, as well as (poultry meat) consumption inevitably lead to the high demand for chicken/poultry meat by countless food services and associated processing industries [5]. Compared to red meat, chicken/poultry meat remains attractive given its high nutritive value, easier-to-handle cuts, and fewer associated religious restrictions [6,7]. Specifically, the proportion of unsaturated fatty acids, readily available protein, low energy content, as well as reasonable amounts of potassium, magnesium, zinc, and B group vitamins strengthens the nutritive value of broiler chicken meat [5]. Following meat and meat products and consumer preferences for poultry meat products [1,2,3], both the carcass characteristics [5,6], and (product) storage performance after various processing strategies [7,8,9,10], equally deserve full attention. Moreover, accelerated postmortem glycolysis is among the meat science tenets that bring about pale, soft, and exudative challenges in poultry meat [8]. The oxidation of polyunsaturated fatty acids (PUFA) in meat products either at cooking, digestion, or storage would facilitate a quality deterioration via advances of lipid oxidation end-products, and the formation of toxic compounds [9]. To avert this challenge, the refrigeration of freshly prepared broiler chicken meat products would reduce both microbial proliferation and lipid peroxidation, particularly during the storage periods. Moreover, seeking better processing and shelf-life extension strategies, for instance, the use of preservatives that help enhance the value of poultry products, are among the key interests of animal product stakeholders [8,10,11,12].

Marination remains among the existing traditional culinary techniques of seasoning. Typically, it involves the soaking of meat products in a slurry/solution that comprises a mix of different ingredients largely equipped with natural bioactive compounds, from vinegar, wine, soy sauce, salt, and herbs, to spices [11,12,13]. Marination processes vary across countries/regions, wherein consumers and other stakeholders apply them to various animal meat products largely directed for the enhancement of both moistness and sensory values, as well as for the provision of tenderness and other refinements to the texture [11,12,13,14]. Specifically, the bioactive compounds present in marinades exert antioxidant as well as antimicrobial potentials, which when applied to animal meat products, cumulatively enhance both the nutritional value and sensory attributes. Oftentimes, marinades would be applied alongside various seasonings with the aim to significantly influence the animal meat product’s flavor development [12,13,15,16]. Indeed, herbs and spices provide beneficial/health-promoting phytochemicals, making their usage in food preservation increasingly important even in recent years [10,17]. More so, marinating ingredients can vary, with examples such as salt, peanut, ginger, black/regular pepper, cranberry pomace, Baikal skullcap (BS), etc. [9,18,19,20,21,22]. Salt enhances the flavor and tenderness, which provides antimicrobial and preservative activity for the meat [14,15]. Peanut (*Arachis hypogaea*) skin constitutes phenolics and other health-promoting compounds with its extract able to deliver total antioxidant activity believed to linearly corroborate the total phenolic concentration [21]. Ginger *(Zingiber officinale*) roots provide functional volatile oil derivatives, phenols, and flavonoids [23], with its extract able to deliver antioxidant properties [18]. Pepper serves important culinary purposes, and common examples include black, white, and green pepper types [9]. Black pepper (*Piper nigrum*), enriched with phenolic compounds, is often employed in meat preparation [9,22]. As a byproduct of cranberry processing with beneficial polyphenols, cranberry pomace is frequently discarded despite the extracts from its seeds, skins, and stems serving as a food ingredient [19]. Herbs such as Baikal skullcap (*Scutellaria baicalensis* L.), can provide antimicrobial effects to dairy products [20]. Moreover, there exists pickle making that involves vinegar/edible oil, salt, spices, and other condiments, which have been understood to offer a high nutritious value to poultry meat [24].

The pursuit of healthy living by consumers is among the crucial rationales that corroborate the increased demand for freshly prepared poultry meat, which associates strongly with their focus on nutritional improvement, not only in Poland but across Europe [2]. Whilst the combined effect of different marinades when applied to poultry meat helps to tenderize the muscle, extend the shelf time, and enhance the consumer appeal [9,11,12,18], the application of thermal treatment remains mandatory in actualizing an increased digestibility, decreased microbial proliferation, and enhanced flavor/texture [25]. In particular, thermal food processing has advanced even more in the recent decades, and examples can range from cook–chill, grilling, sous-vide, aseptic processing, and ohmic heating, to laser-based packaging [26]. Of interest to the authors of this current work is grilling, which depicts a cooking process/type that involves a significant amount of direct/radiant dry heat transferred by conduction [27,28]. When applied to animal meat products, this cooking process/type specifically confers a distinctive aroma and flavor that emanates from the Maillard reaction—a chemical process largely associated with temperatures higher than 155 °C (310 °F). Direct/radiant heat, such as those from a typical oven grill, capably delivers relatively high temperatures to reduce the cooking time of any given meat slice [27,28], as well as facilitate the loss of its fat and juiciness [29]. The charcoal type of grill appears relatively common, and is believed to prepare a chicken breast meat sample in 20 min [30]. Largely considered to be healthier than the charcoal type, oven-grilling remains a useful food process approach increasingly of research interest, particularly its application for animal/meat food products [31,32,33]; however, published information specific to the application of oven-grilling to different marinated chicken breast meat is scarce, to the best of our knowledge. Thus, further exploration specific to this research direction is warranted so as to enhance its consumer appeal and product development. Therefore, this current work investigated the antioxidant, organoleptic, and physicochemical changes in different marinated oven-grilled chicken breast meat. Specifically, the chicken breast meat samples were procured from a local retailer in Wroclaw, Poland. Additionally, the different marination variants involved cranberry pomace (CP), grape pomace (GP), and Baikal skullcap (BS), which subsequently incorporated either African spice (AS) or industrial marinade/pickle (IM).

## 2. Materials and Methods

### 2.1. Schematic Overview of Experimental Program

**Figure 1:**
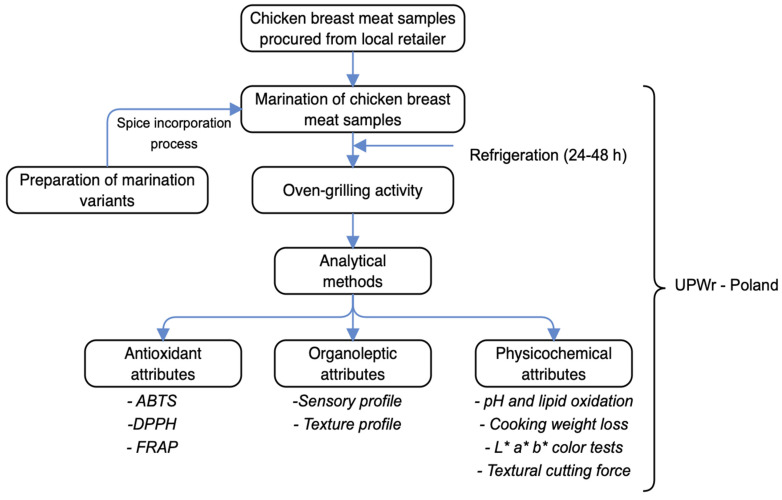
The schematic overview of the experimental program, which depicts the major stages, from the procurement of the chicken breast meat samples, preparation of marinade variants, through oven-grilling activity, and the subsequent analytical measurements. ABTS: 2,2′-Azinobis-(3-ethylbenzthiazoline-6-sulphonate); DPPH: 1,1-diphenyl-2-pierylhydrazy (radical scavenging activity); FRAP: ferric-reducing antioxidant power; UPWr: Uniwersytet Przyrodniczy we Wrocławiu (Wroclaw University of Environmental and Life Sciences, Poland).

The schematic overview of the experimental program, which depicts the major stages, from the procurement of the chicken breast meat samples, the preparation of marinade variants, through the oven-grilling activity, and subsequent analytical measurements, is shown in Figure 1. For emphasis, this work sought to understand the effects oven-grilling applied to chicken breast meat samples subject to different marination variant increments of BS, CP, and GP concentrations that subsequently incorporated either AS or IM, would deliver with respect to antioxidant, organoleptic, and physicochemical changes. Specifically, the antioxidant properties involved 2,2′-azinobis(3-ethylbenzothiaziline-6-sulfonate) (ABTS), 1,1-diphenyl-2-pierylhydrazy (DPPH), and ferric-reducing antioxidant power (FRAP). The organoleptic properties involved a sensory profile by way of flavor, appearance, tenderness and taste, and textural profile by way of hardness, chewiness, gumminess, graininess and greasiness. The physicochemical properties involved pH, thiobarbituric acid reactive substance (TBARS), cooking weight loss, L* a* b* color, and textural cutting force. The chemicals and reagents employed in this study were of an analytical grade standard. All the conducted analytical measurements involving different marinated oven-grilled samples were independently performed consistent with the relevant guidelines set out by the Department of Functional Food Products Development, Wroclaw University of Environmental and Life Sciences, Poland.

### 2.2. Procurement, and Storage of Chicken Breast Meat Samples

The chicken breast meat samples (~20 kg) were procured from a local retail food distributor that serves the Wroclaw region, and immediately after, transported to the Department of Functional Food Products Development, Wroclaw University of Environmental and Life Sciences, Poland. Upon arrival, the chicken breast meat samples were rapidly stored in cold room refrigeration (~4 °C), and from there, made available for the marination and oven-grilling processes.

### 2.3. Preparation of Marinades, and Marination Variants/Process

The preparation of the marinades involved salt (1.6 g), together with incremental 0.5, 1, and 1.5% constituent quantities of ground BS, CP, and GP been representative of additive antioxidants. To initiate a new product development perspective to this study, either African spice (AS) or industrial marinade/pickle (IM) (each constituting 4 g) were incorporated. Specifically, the African spice (Fresh and Tasty Kebab Powder) procured from Fresh and Tasty Farms Ltd. (Accra-North, Ghana) and prepared in adherence to the quality standards set by the Food and Drugs Authority (FDA) Ghana, comprised such ingredients as peanut and ginger, as well as black/regular pepper. This specific AS product was selected given its growing popularity and usage in barbecues in Wrocław (as well as other places in Poland). Further, the industrial marinade/pickle (Marinate do mięs) procured from Regis(R) Food Technology (Regis sp. z o.o., Kraków, Poland), whose preparation adhered to the quality standards of the International Organization for Standardization (ISO), British Retail Consortium (BRC), and International Food Standard (IFS), comprised such ingredients as thyme, oregano, rosemary, marjoram, and parsley. Additionally, this specific IM product was selected given its growing market and reputation not only in Poland, but also in other parts of the EU.

As mentioned earlier, the incremental constituent quantities of BS, CP and GP, which thereafter incorporated either AS or IM, resulted in the marination variants implemented as follows: (1) the control (antioxidant additive % = 0.0); (2) the control (antioxidant additive % = 0.5); (3) the control (antioxidant additive % = 1.0); (4) the control (antioxidant additive % = 1.5); (5) AS (antioxidant additive % = 0.0); (6) AS (antioxidant additive % = 0.5); (7) AS (antioxidant additive % = 1.0); (8) AS (antioxidant additive % = 1.5); (9) IM (antioxidant additive % = 0.0); (10) IM (antioxidant additive % = 0.5); (11) IM (antioxidant additive % = 1.0); and (12) IM (antioxidant additive % = 1.5). The immersion method as described by Sokołowicz et al. [12] with slight modifications was followed to implement the marination process of the chicken breast meat samples. This required the use of plastic containers approved for contact with food and a 1:2 ratio that applied to the weight of the meat (g) and marinade volume (mL). The chicken breast meat samples were dipped sufficiently in the marinade variants for a 24 h period at ~4 °C. When the marination immersion time had been completed, the samples were allowed to drain for 5 min, placed in folded foiled packages, and were then ready for the oven-grilling activity.

### 2.4. Oven-Grilling Activity

The oven-grill activity, consistent with the method modified from Salmon, Knize, and Felton [16], was applied to the various marinated chicken breast meat samples. The oven-grilling process employed a commercially available electric hot air convection type facility (CAMRY CR 6017, Serwis Centralny Camry, Warszawa, Poland) that operated with 2200 W power, and a set temperature of 180 °C. The chicken breast meat samples were placed evenly-spaced on a grill rack of the pre-heated oven, which had its heat setting set to move evenly from the bottom and from the top. The oven-grill facility remained closed during the cooking process, and would only be opened to either remove or place the samples. The internal temperatures of the chicken breast meat samples were routinely checked to keep them at roughly 75 °C. The cooking time was kept constant at 5 min, and this was applied to all the marinated samples in this study. After the oven-grill process had been completed, the chicken breast meat samples were allowed to briefly cool for 15 min, and then subsequently refrigerated (~4 °C) during which the samples were analyzed.

### 2.5. Analytical Measurements 

#### 2.5.1. Determination of Antioxidant Aspects

The 2,2′-azinobis(3-ethylbenzothiaziline-6-sulfonate) (ABTS) radical scavenging activity was determined following method described by Bai et al. [34] with a slight modification. Briefly, an already prepared ABTS^+^ solution was diluted with ethanol. Subsequently, 990 μL of the ABTS^+^ solution was added to 10 μL of a meat tissue supernatant, which was followed by incubation at ambient temperature (~25 °C) for 6 min. The blank comprised 990 μL of the ABTS^+^ solution mixed with 10 μL of EtOH 70%. The absorbance was read against a blank at 734 nm using a UV-Vis Spectrophotometer (GENESYS™ 180, ThermoFisher Scientific Inc., Waltham, MA, USA), and the ABTS^+^ radical scavenging activity was presented in mM Trolox.

The 1,1-diphenyl-2-pierylhydrazy (DPPH) radical scavenging activity was determined following method described by Zhang et al. [22] with a slight modification. The ethanolic DPPH radical solution (4 mg of DPPH in 100 mL of 95% ethyl alcohol) was freshly prepared. Briefly, aliquots (20 μL) from the meat tissue supernatant were vigorously mixed with 200 μL 0.3 mM of the ethanolic DPPH radical solution, by vortex for 1 min, and subsequently left to stand at ambient temperature (~25 °C) for 30 min in the dark. After incubation, the reduction of the DPPH was read at 517 nm using a UV-Vis Spectrophotometer (GENESYS™ 180, ThermoFisher Scientific Inc., Waltham, MA, USA) against a blank (1.5 mL of the DPPH solution and 1 mL of 95% ethanol). The DPPH radical scavenging activity was expressed in mM Trolox.

The ferric-reducing antioxidant power (FRAP) was determined as slightly modified from Lengkidworraphiphat et al. [35]. Ethanol extracts of chicken breast meat samples were prepared using 70% EtOH. The FRAP solution was comprised of 10 mM of 2,4,6-tripyridyl-s-triazine (TPTZ) and 20 mM of ferric chloride, added with 300 mM of a sodium acetate buffer (pH 3.6), at a ratio of 1:1:10 (*v*:*v*:*v*), which was incubated for 30 min at 37 °C. The blank was comprised of 3 mL of the FRAP reagent mixed with 1 mL of EtOH. The absorbance was read at 593 nm against the blank using a UV-Vis Spectrophotometer (GENESYS™ 180, ThermoFisher Scientific Inc., Waltham, Massachusetts-USA). The FRAP value of each sample was expressed as mM/dm^3^.

#### 2.5.2. Determination of Organoleptic Aspects

The organoleptic aspects of the various marinated oven-grilled chicken breast meat samples comprised sensorial analysis modified from Augustyńska-Prejsnar, Ormian, and Sokołowicz [36], and textural profiling modified from Sanchez Brambila, Bowker, and Zhuang [37]. The sensory panelists comprised ten (N = 10) staff and graduate students of the Department of Functional Food Products Development, Wrocław University of Environmental and Life Sciences (Wrocław-Poland). All panelists, already familiar with the evaluation criteria laid out specifically for this current study, were particularly required to discriminate between the levels of chicken breast meat’s flavor, appearance, tenderness, taste, and off-flavor for the sensorial analysis, as well as the hardness, chewiness, gumminess, and graininess for the textural profiling. In addition to verbal consent taken prior to the organoleptic evaluation, and with the panelists’ participation being voluntary, no names/genders were reported to ensure privacy. The organoleptic evaluation took place in a well-ventilated room of neutral color, proper lighting, and distraction-free. To perform the sensory and texture profile assessment, the evenly cut samples already cooled to 20 ± 2° C were placed in coded white plastic plates in triplicates. Consistent with Çakmakçı et al. [38], each panelist used warm water to cleanse their taste palates between samples, to ensure the previous evaluation did not affect the (taste of the) new one. Each panelist simultaneously evaluated the coded samples using a five-point scale (1 point being the lowest score and 5 points being the highest) for the sensory aspects, and using a 0 to 15 intensity scale for the texture profile, as adapted from Civille and Thomas Carr [39].

#### 2.5.3. Determination of Physicochemical Aspects

The pH measurement was determined as slightly modified from Barido and Lee [40], specifically conducted before and after the oven-grilling activity. This required a 5 g sample mixed with 45 mL of distilled water using a homogenizer (Model PH-91, SMT Company, Chiba, Japan) at 10,000 rpm, for 1 min. The portable pH meter (HI 99,163 Hanna Instrument Company, Vöhringen, Germany) was technically calibrated using buffer solutions (with an approximate pH of 4.0, 7.0 and 9.0) at ambient conditions. The variations in pH were elicited by a difference between those of the oven-grilled samples and the control.

The thiobarbituric acid reactive substance (TBARS) was determined as slightly modified from Luciano et al. [41], specifically conducted before and after the oven-grilling process. With the help of a stomacher, the chicken breast meat samples (1.0 g) were homogenized with 10 mL of 10% trichloroacetic acid (TCA) for 1 min to precipitate the proteins present in the meat. Next, there was a centrifugation at 4000× *g* (MPW-351R refrigerated, MPW Med. Instruments, Warszawa, Poland), after which the emergent mix was subject to filtration (Whatman #1 filter paper), then 2 mL of the supernatant was transferred to 2 mL of 0.06 M thiobarbituric acid. The reaction mixture was kept in a water bath at 100 °C for 40 min, followed by cooling in an ice-water bath (~2 min). The calibration curve was prepared using 1,1,3,3-tetra-ethoxypropane in TCA, as a standard solution. The samples were finally analyzed, with the absorbance read at 532 nm using a UV-Vis Spectrophotometer (GENESYS™ 180, ThermoFisher Scientific Inc., Waltham, MA, USA). According to the standard curve equation, the TBARS values were reported as mg of malondialdehyde (MDA) per kg of meat sample.

The cooking weight loss was determined as slightly modified from Ali et al. [42]. Specifically, the samples were weighed prior to and after the oven-grilling. The cooking weight loss depicted the cooked sample (B) weight as a percentage of the precooked sample (A) weight, as shown by the Equation (1) below:

Cooking loss (%) = [(A − B)/(A)] × 100(1)

The color measurements were determined as slightly modified from Kopec et al. [43], specifically being conducted before and after oven-grilling by way of the CIE L* a* b* scale (L* = darkness; a* = redness/greenness; and b* = yellowness/blueness) using a Minolta CR-40 reflection colorimeter (Konica Minolta Sensing Europe B.V., NL-3439 MR, Nieuwegein, The Netherlands). Three individual measurements were taken of different areas on the chicken breast meat surface from each treatment group, and the results via the CIE L* a* b* colorimetric system were displayed in real-time.

The textural cutting force was determined as slightly modified from Augustyńska-Prejsnar, Ormian and Sokołowicz [44]. This required measuring the force that was necessary to cut a piece of a chicken breast meat sample. A Zwick/Roell testing machine (Zwick GmbH & Co. KG, Ulm, Germany), equipped with a Warner-Bratzler V-blade knife, was employed to measure the cutting force (Fmax) at a head speed of 100 mm/min and an initial force of 0.2 N. Three different chicken breast meat samples from each treatment group, with respective estimated cross-sectional diameters and lengths of 100 mm^2^ and 50 mm, were subjected to textural cutting force measurements.

### 2.6. Statistical Analysis 

The resultant data independently generated from the different samples using a minimum of two determinations, with a few exceptions, were given to a one-way analysis of variance (ANOVA). The results were represented as a mean ± standard deviation (SD). The probability level was statistically significant at *p*  <  0.05 (95% confidence level). The Statistica 13.0 software (StatSoft GmbH, Hamburg Germany) was used to run the data.

## 3. Results and Discussion

### 3.1. Changes in Antioxidant Properties

**Figure 2:**
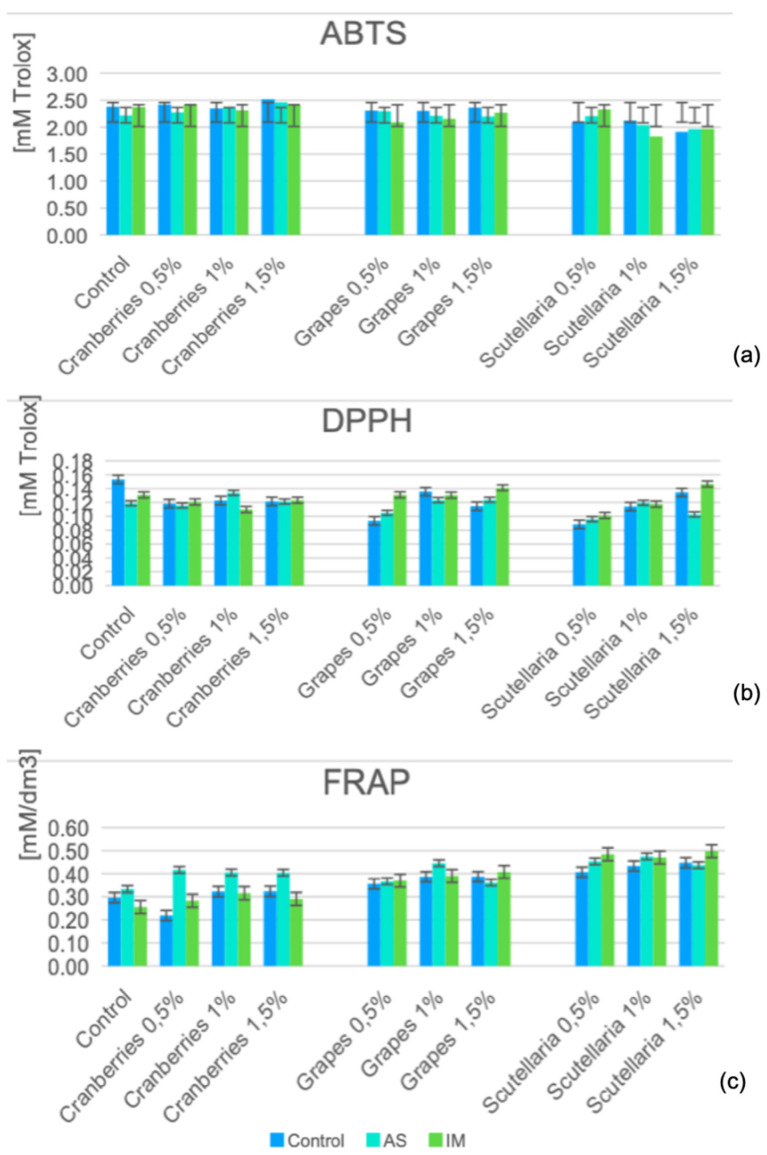
Changes in (**a**) ABTS, (**b**) DPPH, and (**c**) FRAP values across the various marinated oven-grilled chicken breast meat samples compared to a control. The marination variants involved cranberry pomace (“Cranberries” in Figure), grape pomace (“Grapes” in Figure) and Baikal skullcap (“Scutellaria” in Figure) that incorporated either African spice (AS) or industrial marinade/pickle (IM). Results are expressed as mean ± standard deviation (SD) at the probability level of *p* < 0.05. ABTS: 2,2’-Azinobis-(3-ethylbenzthiazoline-6-sulphonate); DPPH: 1,1-diphenyl-2-pierylhydrazy (radical scavenging activity); FRAP: ferric-reducing antioxidant power.

Changes in the ABTS, DPPH, and FRAP values across the various marinated oven-grilled chicken breast meat samples compared to the controls can be seen in Figure 2. Overall, some statistical differences (*p* < 0.05) in the ABTS, DPPH, and FRAP occurred across the various marinated oven-grilled chicken breast meat samples either before or after incorporating the AS or IM, with minimum (ABTS = 1.82 ± 0.11 mM Trolox at IM + GP 1.5%; DPPH = 0.09 ± 0.00 mM Trolox at *Scutellaria* 0.5%; FRAP = 0.26 ± 0.07 mM/dm^3^ at AS + CP 1%) and maximum(ABTS = 2.52 ± 0.21 mM Trolox at AS + *Scutellaria* 1%; DPPH = 0.15 ± 0.00 mM Trolox at IM + *Scutellaria* 1.5%; FRAP = 0.50 ± 0.19 mM/dm^3^ at IM + *Scutellaria* 1.5%) values. Specifically, prior to incorporating either the AS or IM, both the ABTS and FRAP ranges appeared somewhat limited across the control samples of the CP, GP, and BS (ABTS = from 2.09 ± 0.02 to 2.36 ± 0.23 mM Trolox; FRAP range = from 0.30 ± 0.01 to 0.45 ± 0.02 mM/dm^3^). However, applying either the AS or IM appeared to widen the ABTS and FRAP ranges of the various marinated oven-grilled chicken breast meat samples (specific to the AS, the ABTS range: from 2.22 ± 0.10 to 2.52 ± 0.21 mM Trolox; FRAP range = from 0.26 ± 0.07 to 0.41 ± 0.03 mM/dm^3^; and specific to the IM, the ABTS range: from 1.82 ± 0.11 to 2.42 ± 0.06 mM Trolox; FRAP range = from 0.32 ± 0.04 to 0.50 ± 0.19 mM/dm^3^), but not quite those of the DPPH. Moreover, the ABTS, DPPH, and FRAP values across the CP, GP and BS concentration increments seemingly likened those when either the AS or IM were incorporated. Probably, the individual/collective impact of the constituent ingredients that made up either the AS or IM contributed to the antioxidative efficacy.

Further, the ABTS, DPPH, and FRAP values would not necessarily align with either the CP, GP, or BS concentration increments despite their individual antioxidant potentials. For example, both Istrati et al. [11], and Shahidi and Hossain [17] considered herbs/spices with very promising antioxidant and antimicrobial properties, coupled with the acidic or alkaline nature of their solutions, poised to enhance the shelf quality of any given animal product. Additionally, measurements of ABTS, DPPH, and FRAP assays are increasingly frequent when evaluating the antioxidant activity of plant-oriented marinades/spices [10,22,35]. Notably, an ABTS assay would detect the generation of an (ABTS^+^) radical, which is considered to be in a stable form prior to a reaction with antioxidants. Further, a DPPH assay would detect the capacity of antioxidants to decolorize a radical solution, which would suggest the lipophilic antioxidant level of a given food sample [45]. Contextualizing both ABTS and DPPH assays, Floegel et al. [45] considered both cranberry and grapes among the food items with a promising top total antioxidant capacity (TAC). Moreover, Sáyago-Ayerdi et al. [46] understood the GP composition to comprise concentrates of grape seeds, stems, and peel, which are enriched with phenolic compounds. Additionally, Kim et al. [47] considered the ethanol extracts of Baikal skullcap (BS) among the medicinal herbs with an antimicrobial potential, given the presence of amino acids, essential oils, flavones, and phenylethanoids, as well as sterols, whereas Lee et al. [48] considered the dried roots of BS to possess ample amounts of flavonoids.

Considering that this current study had no storage period, understanding what brings about the interactive effects that emerge after applying antioxidant additive increments of either CP, GP, or BS concentrations alongside AS or IM for the various marinated oven-grilled chicken breast meat samples appear challenging. By studying whether the turmeric and black pepper spices were able to decrease the lipid peroxidation in meat patties, Zhang et al. [22] understood that the cooking temperatures might not necessarily contribute to the interactive effects (of combined turmeric and black pepper). Meanwhile, the incorporation of either CP, GP, or BS together with either AS or IM should constitute a herb mix with the capacity to increase the antioxidant activity of a given marinade medium, as well as decrease the lipid breakdown [22,25], and applying heat temperatures especially above 120 °C such as those with oven-grilling should capably decrease the antioxidant activity by breaking the primary compounds within the tissues (of a herb mix) [40]. By increasing the liberation of flavonoid and polyphenol bonds, such heat temperatures above 120 °C would further deactivate the endogenous oxidative enzymes, which could deter the availability of the antioxidative compound that would generate a more stable product [40].

### 3.2. Changes in Organoleptic Properties

Sensory evaluation is considered among the increasingly popular approaches employed in evaluating the freshness of marinated chicken meat products, given the fast, immediate, and simple information it provides regarding the product quality [49]. Herein, changes in the sensory profile by way of the flavor, appearance, tenderness, and taste, and the textural profile by way of the hardness, chewiness, gumminess, graininess, and greasiness of the various marinated oven-grilled chicken breast meat samples, are respectively shown in Table 1 and Table 2. With the exception of the sensorial tenderness, as well as the textural chewiness, gumminess, and greasiness, there were resemblances (*p* > 0.05) in the organoleptic properties across the different marinated oven-grilled chicken breast meat samples, with promising ranges in the sensorial flavor (from 3.50 ± 1.28 to 4.31 ± 1.20), appearance (from 3.50 ± 0.60 to 4.25 ± 0.89), tenderness(from 3.19 ± 0.71 to 4.50 ± 0.76), taste (from 3.17 ± 0.95 to 4.31 ± 0.74), and flavor (from 4.00 ± 1.07 to 5.00 ± 1.41), and the textural hardness (from 2.88 ± 1.13 to 5.00 ± 1.51), chewiness (from 2.38 ± 1.30 to 4.25 ± 2.12), gumminess (from 2.00 ± 1.31 to 4.25 ± 1.49), graininess (from 2.25 ± 1.06 to 3.38 ± 1.25), and greasiness (from 1.56 ± 0.92 to 4.88 ± 2.70). Specifically, some fluctuating organoleptic values seemed apparent with increasing CP, GP, and BS concentrations. Incorporating either the AS or IM alone produced sensory scores of the flavor, appearance, tenderness, taste, and off-flavor that were likened with those of the CP, GP, and BS. Further, the textural hardness scores appeared seemingly higher for the GP, whereas this was true for the greasiness for the CP, and gumminess for the BS.

**Figure 3:**
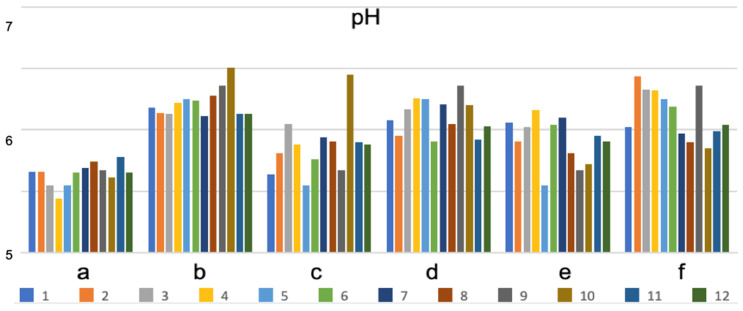
Changes in pH across the various marinated chicken breast meat samples before and after oven-grilling. The different letters (shown along the *x*-axis) represent as follows: (a) CP before oven-grill; (b) CP after oven-grill; (c) GP before oven-grill; (d) GP after oven-grill; (e) BS before oven-grill; (f) BS after oven-grill. The numbers representing the different color shades are as follows: (1) control (antioxidant additive % = 0.0); (2) control (antioxidant additive % = 0.5); (3) control (antioxidant additive % = 1.0); (4) control (antioxidant additive % = 1.5); (5) AS (antioxidant additive % = 0.0); (6) AS (antioxidant additive % = 0.5); (7) AS (antioxidant additive % = 1.0); (8) AS (antioxidant additive % = 1.5); (9) IM (antioxidant additive % = 0.0); (10) IM (antioxidant additive % = 0.5); (11) IM (antioxidant additive % = 1.0); (12) IM (antioxidant additive % = 1.5). African spice: AS; industrial marinade/pickle: IM; CP: cranberry pomace; GP: grape pomace; BS: Baikal skullcap.

**Figure 4:**
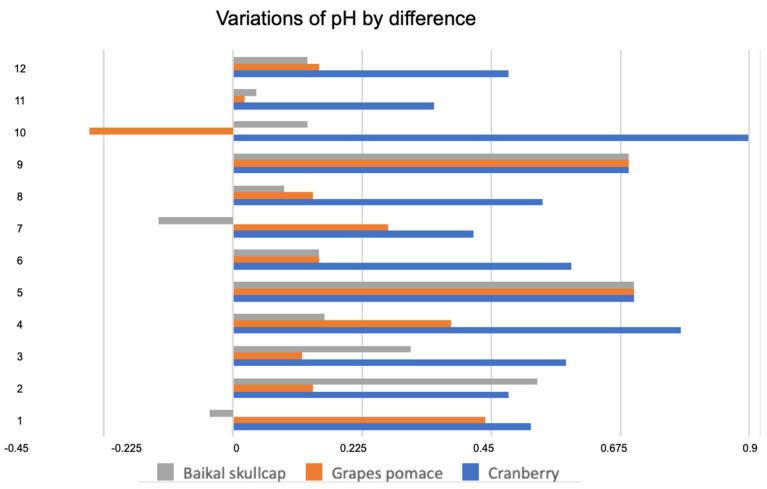
Variations of pH by difference across the various marinated oven-grilled chicken breast meat samples. The numbers (shown along the *y*-axis) represent as follows: (1) control (antioxidant additive % = 0.0); (2) control (antioxidant additive % = 0.5); (3) control (antioxidant additive % = 1.0); (4) control (antioxidant additive % = 1.5); (5) AS (antioxidant additive % = 0.0); (6) AS (antioxidant additive % = 0.5); (7) AS (antioxidant additive % = 1.0); (8) AS (antioxidant additive % = 1.5); (9) IM (antioxidant additive % = 0.0); (10) IM (antioxidant additive % = 0.5); (11) IM (antioxidant additive % = 1.0); (12) IM (antioxidant additive % = 1.5). African spice: AS; and industrial marinade/pickle: IM.

**Figure 5:**
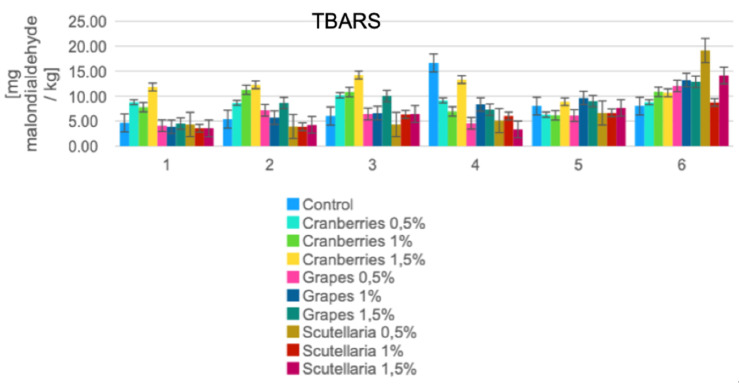
Changes in thiobarbituric acid reactive substance (TBARS) across the various marinated oven-grilled chicken breast meat samples. Results are expressed as mean ± standard deviation (SD) at the probability level of *p* < 0.05. The numbers (shown along the *x*-axis) represent as follows: (1) control before oven-grill; (2) AS before oven-grill; (3) IM before oven-grill; (4) control after oven-grill; (5) AS after oven-grill; (6) IM after oven-grill; African spice: AS; and industrial marinade/pickle: IM.

**Figure 6:**
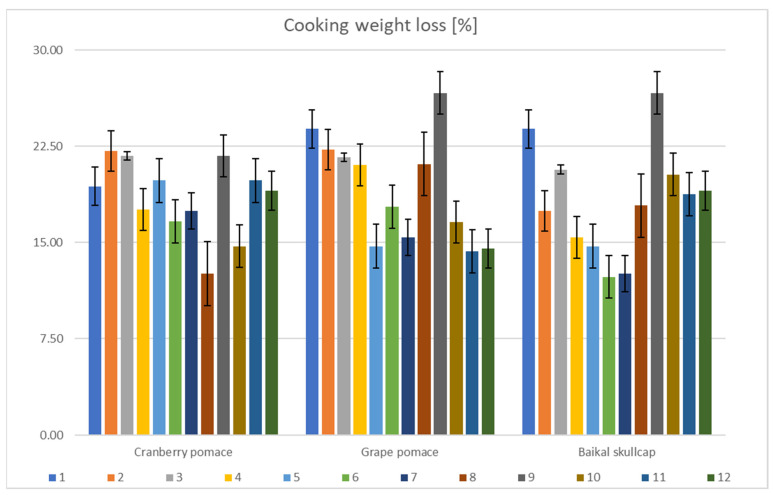
Changes in cooking weight loss (%) across the various marinated oven-grilled chicken breast meat samples compared to the control. Results are expressed as mean ± standard deviation (SD) at the probability level of *p* < 0.05. The numbers representing different color shades are as follows: (1) control (antioxidant additive % = 0.0); (2) control (antioxidant additive % = 0.5); (3) control (antioxidant additive % = 1.0); (4) control (antioxidant additive % = 1.5); (5) AS (antioxidant additive % = 0.0); (6) AS (antioxidant additive % = 0.5); (7) AS (antioxidant additive % = 1.0); (8) AS (antioxidant additive % = 1.5); (9) IM (antioxidant additive % = 0.0); (10) IM (antioxidant additive % = 0.5); (11) IM (antioxidant additive % = 1.0); (12) IM (antioxidant additive % = 1.5). African spice: AS; and industrial marinade/pickle: IM.

Generally, consumer perceptions and responses to organoleptic textures would vary, for instance, in the attributes associated with tenderness [50]. Whereas the human senses altogether would help in ascertaining the overall acceptability by detecting such sensory properties as the appearance, flavor, taste, and texture, a descriptive sensory analysis should provide trained panels the capacity to discriminate different/diverse sensory aspects [51] of any given tested animal food products. In the current study, for example, a higher textural hardness score could occur when the AS was incorporated, particularly for the BS of different marinated oven-grilled chicken breast meat samples. Conversely, as the IM was incorporated, higher textural chewiness scores could occur either for the GP, or greasiness scores for the CP, or hardness scores for the BS. Given the somewhat limited range values, establishing the specific organoleptic sensory and texture profile trends across the various marinated oven-grilled chicken breast meat samples of this study appear challenging (Table 1 and Table 2). This might corroborate the somewhat limited ranges of both the antioxidant and physicochemical outcomes observed at other sections of this work, especially where those of increasing CP, GP, and BS concentrations likened with those incorporating either AS or IM (Refer to Figure 2, Figure 3, Figure 4, Figure 5 and Figure 6 and Figure 7 in Ref, and Table 3). The numerical texture data obtained from instrumental measurements corroborated the sensory results. Additionally, instrumental texture would corroborate the sensory tenderness acceptability, as evidenced in chicken breast meat reported elsewhere [52], and such a sensory-texture connection should be obtainable given the highly cross-linked nature of collagen that would be elevated at the post-slaughter stage, which eventually toughens a poultry chicken meat product [53]. Largely, such factors such as the connective tissue and cross-linking, intramuscular fat (IMF), myofibrillar integrity, and protein denaturation during cooking, as well as the sarcomere length would influence meat tenderness [50].

### 3.3. Changes in Physicochemical Properties

Changes in the pH, TBARS, cooking weight loss, L* a* b* color, and textural cutting force across the various marinated oven-grilled chicken breast meat samples compared to the control, respectively, are shown in Figure 3, Figure 4, Figure 5 and Figure 6 and Table 3 and Table 4. Increasing either the CP, GP, or BS concentrations produced varying pH, TBARS, cooking weight loss, L* a* b* color, and textural cutting force values, which in many instances could not demonstrate a distinct trend. For instance, without incorporating either AS or IM, the pH showed somewhat limited ranges, such as the CP pre-oven grill (from 5.44 to 5.78), CP post-oven grill (from 6.11 to 6.51), GP pre-oven grill (from 5.55 to 6.45), GP post-oven grill (from 5.91 to 6.36), BS pre-oven grill (from 5.55 to 6.16), and BS post-oven grill (from 5.85 to 6.44) (Figure 3). A greater variation of pH by difference seemed so at the concentration increments of the CP, slightly less at GP, followed by BS (Figure 4). In studying the antimicrobial/antioxidant activities of spice extracts on raw chicken meat quality, Zhang, Wu and Gun [10] reported an initial pH of 5.65 ± 0.05 at the beginning of the storage period. Associated with pH increases could be the accumulation of ammonia, and the utilization of amino acids by bacteria being released during protein degradation. Moreover, antimicrobial ingredients in natural spices could provide some inhibitory effects that could be associated with pH decreases [10]. Additionally, herbs/plants that contain a high antioxidant capacity should capably prevent pH increases. Consequently, the pH range values of the various marinated oven-grilled chicken breast meat samples might corroborate the widely-accepted post-rigor value range of 5.90–6.10 [40].

Typically, at the post-slaughter stage of any given broiler chicken, a cascade of lipid peroxidation events takes place and continues even after the application of thermal processing (on the resultant carcass/meat) [54]. At slaughter, the interruption of the blood flow specifically halts the metabolic processes, which results in the development of oxidative rancidity that directly cumulates to the catalyzed phospholipids via heme proteins [54]. Figure 5 shows the various marinated oven-grilled chicken breast samples that obtained a wide range of TBARS values, both before (with minimum TBARS = 3.55 ± 0.13 MDA/kg of the control BS at 1 and 1.5%; maximum TBARS = 12.27 ± 0.00 MDA/kg of the AS + CP 1.5%) and after oven-grilling (with minimum TBARS = 3.32 ± 0.06 MDA/kg of the control BS at 1.5%; maximum TBARS = 12.27 ± 0.00 MDA/kg of the IM + BS 1.5%). Figure 5 also shows that the oven-grilling process momentarily increased the TBARS values of some of the marinated chicken breast meat samples, despite the fluctuating values when both the AS and IM were incorporated. Compared to raw meat, pre-prepared meat products, particularly those submitted to thermal processing, are considered more susceptible to lipid oxidation [54]. Higher temperatures from thermal processing such as with oven-grilling probably progress the release of oxygen, heme, and iron, which by inducing the production of free radicals subsequently kickstart the development of undesirable off-odors/flavors [54]. The process of lipid peroxidation generates oxygen free radicals that decrease food quality and shelf-time, which would require the promising effects of antioxidants, especially those capable of suppressing oxidative stress [34]. Thus, incorporating such marinade variants as either the BS, CP, or GP concentrations reported herein should capably provide available antioxidant potentials. For instance, GP, whether from the contexts of extractable (anthocyanins, flavonols, flavan-3-ols, and phenolic acids) and non-extractable polyphenols (polymeric proanthocyanidins and high molecular weight hydrolyzable tannins), should have the capacity to scavenge radicals, and help prevent the onset of rancidity [55].

Figure 6 shows that the cooking weight loss varied considerably between a minimum of ~12.31% (AS + BS 1.0% conc.) and a maximum of ~26.67% (IM alone). Compared to the control, incorporating AS reduced the cooking weight loss of the various marinated oven-grilled chicken breast samples, but much less for the IM. The degree of cooking weight loss, dependent on the cooking time and the temperature band, further underpins the potential of oven-grill heat temperatures to not only expel the moisture thereof, but more so to modify the textural properties [28]. In the various marinated oven-grilled chicken breast meat samples herein, the cumulative impact of all the (above-mentioned) detected lipid oxidation (TBARS), cooking weight loss, and pH differences would have likely influenced the color [42,47]. Table 3a–c shows that, without incorporating either AS or IM, the L* a* b* color of the various oven-grilled chicken breast meat samples differed significantly (*p* < 0.05) with the BS, CP, and GP concentration increments. The L* a* b* color obtained varying ranges, from ~42.56 to ~83.30 at L*, from ~2.55 to ~5.80 at the a*, and from ~2.36 to ~29.05 at the b* color scales. Further, incorporating either the AS or IM continued to produce significant differences (*p* < 0.05) in the L* a* b* color values. Potentially, the oven-grilling activity contributed to pushing the L* a* b* color of the various marinated chicken breast meat samples herein towards the extreme values, either negatively or positively. Adding together the differences in the connective tissues, alongside the ability of myofibrillar and soluble proteins to bring about textural changes [56], when the heat temperatures such as those coming from an oven-grill facility exceeding the 100 °C mark are applied to an animal meat product, an irreversible denaturation of the heme proteins (i.e., hemoglobin and myoglobin) that likely initiate structural changes can occur, which might result in lightening the color [57]. 

In Table 4, the incorporation of either AS or IM, together with either increased CP, GP, or BS concentrations to form a herbal mix, produced textural cutting force fluctuations that showed several trends. For instance, incorporating the AS with either increased CP, GP, or BS concentration(s) appeared to initially reduce the cutting force compared to the control, which may well have been stabilizing the fluctuation; however, incorporating the IM with either increased CP, GP, or BS concentration(s) could have produced an initial greater cutting force, with more for the CP, which seemingly represented an increasing trend. Istrati et al. [11] understood that marination could potentially decrease a meat muscle’s adhesiveness, chewiness, cohesiveness, hardness, and springiness, and at the same time, increase that of its tenderness, regardless of the applied method and ageing. Sokołowicz et al. [12] believed that, besides the brightness of (chicken breast) meat to corroborate the pH values, the sour nature of marinades and the overall impact of antioxidants would potentially influence the textural properties.

## 4. Conclusions

This current work has investigated the antioxidant, organoleptic, and physicochemical changes in different marinated oven-grilled chicken breast meat. For emphasis, the chicken breast meat samples were secured from broilers farmed in Poland. Results showed that the CP, GP, and BS concentration increases would not necessarily go along with the ABTS, DPPH, and FRAP despite changes in the pH, cooking weight loss, L* a* b* color and cutting force values, even after incorporating either AS or IM. Establishing specific organoleptic sensory and texture profile trends across increasing CP, GP, and BS concentrations for the various marinated oven-grilled chicken breast meat samples proved challenging. Holistically, this current work provides useful information about some key quality attributes consumers could anticipate in marinated oven-grilled chicken breast meat shortly after preparation/processing. Despite that this current study was unable to delineate the best marinated oven-grilled chicken breast meat, the authors herein are convinced that an oven-grilling approach is promising to moderate the antioxidant, organoleptic, and physicochemical value ranges of various (marinated oven-grilled chicken breast meat) samples. The shelf-life capacity of the oven-grill technique should be the target of future study, which would require subjecting various (oven-grilled) marinated chicken breast meat samples to different packaging and refrigerated storage conditions. Another future work could employ advanced techniques so as to identify the various bioactive compounds present in marinades, which would provide the foundation to further examine/understand their underlying molecular mechanisms that take place when producing oven-grilled marinated chicken breast meat samples.

## Figures and Tables

**Table 1:** Changes in sensory profile by way of flavor, appearance, tenderness, and taste across the various marinated oven-grilled chicken breast meat samples compared to the controls.

			Flavor	Apperance	Tenderness	Taste	Off-Flavor
**Control**	**Control**	**0%**	3.86 ± 0.96^b^	3.83 ± 1.02^b^	3.93 ± 0.82^abc^	3.44 ± 0.62^b^	4.63 ± 0.74^b^
	**Grape pomace**	**0.5%**	3.75 ± 0.46^b^	3.81 ± 0.65^b^	3.56 ± 0.62^abc^	3.31 ± 0.70^b^	4.00 ± 1.07^b^
		**1%**	3.81 ± 0.59^b^	3.50 ± 0.60^b^	3.94 ± 1.02^abc^	3.94 ± 0.68^b^	4.63 ± 0.52^b^
		**1.5%**	3.94 ± 0.86^b^	3.69 ± 0.80^b^	4.00 ± 0.96^abc^	3.88 ± 0.64^b^	4.50 ± 0.76^b^
	**Cranberry pomace**	**0.5%**	4.19 ± 1.04^b^	3.94 ± 0.68^b^	4.31 ± 0.93^bc^	3.38 ± 0.92^b^	4.38 ± 0.76^b^
		**1%**	4.06 ± 0.64^b^	3.88 ± 0.83^b^	4.13 ± 0.68^abc^	4.13 ± 0.52^b^	4.56 ± 0.52^b^
		**1.5%**	3.69 ± 1.24^b^	3.88 ± 0.98^b^	3.69 ± 0.73^abc^	3.69 ± 0.99^b^	4.19 ± 1.04^b^
	***Scutellaria baicalensis***	**0.5%**	3.61 ± 1.02^b^	3.83 ± 1.13^b^	3.67 ± 0.65^abc^	3.17 ± 0.95^b^	4.56 ± 0.62^b^
		**1%**	3.81 ± 0.92^b^	3.50 ± 0.83^b^	3.19 ± 0.71^ab^	3.19 ± 1.07^b^	4.38 ± 0.46^b^
		**1.5%**	3.81 ± 0.82^b^	3.69 ± 0.93^b^	4.25 ± 0.86^bc^	3.63 ± 0.70^b^	4.75± 0.50^b^
**African Spices**	**Control**	**0%**	4.25 ± 0.71^b^	3.88 ± 1.13^b^	4.50 ± 0.76^c^	4.25 ± 0.93^b^	4.13 ± 1.36^b^
	**Grape pomace**	**0.5%**	4.00 ± 0.89^b^	3.94 ± 0.78^b^	4.06 ± 0.50^abc^	4.31 ± 0.46^b^	4.25 ± 1.04^b^
		**1%**	3.94 ± 0.78^b^	3.88 ± 0.83^b^	4.19 ± 0.75^bc^	3.88 ± 0.64^b^	4.25 ± 1.16^b^
		**1.5%**	3.75 ± 1.25^b^	3.75 ± 1.07^b^	3.56 ± 1.18^abc^	3.88 ± 0.99^b^	4.50 ± 0.76^b^
	**Cranberry pomace**	**0.5%**	4.19 ± 0.73^b^	4.25 ± 0.76^b^	4.00 ± 0.64^abc^	4.00 ± 0.83^b^	4.25 ± 1.16^b^
		**1%**	4.19 ± 0.65^b^	4.00 ± 0.92^b^	3.81± 0.75^abc^	3.88 ± 0.86^b^	4.25 ± 0.93^b^
		**1.5%**	3.94 ± 1.08^b^	3.94 ± 1.07^b^	3.88 ± 0.52^abc^	3.94 ± 0.95^b^	4.38 ± 1.07^b^
	***Scutellaria baicalensis***	**0.5%**	3.93 ± 1.35^b^	3.86 ± 1.22^b^	3.93 ± 0.46^abc^	4.00± 1.44^b^	5.00 ± 1.41^b^
		**1%**	3.81 ± 0.65^b^	4.13 ± 1.31^b^	3.75 ± 0.92^abc^	3.81 ± 1.00^b^	4.75 ± 1.06^b^
		**1.5%**	3.94 ± 0.92^b^	4.00 ± 1.03^b^	4.06 ± 0.65^abc^	4.31 ± 0.74^b^	5.00 ± 0.46^b^
**Industrial**	**Control**	**0%**	4.06 ± 1.27^b^	4.25 ± 0.71^b^	4.13 ± 0.83^abc^	4.00 ± 1.07^b^	4.13 ± 1.13^b^
	**Grape pomace**	**0.5%**	4.13 ± 0.99^b^	3.63 ± 0.74^b^	3.69 ± 0.70^abc^	4.06 ± 0.68^b^	4.25 ± 1.16^b^
		**1%**	3.69 ± 1.19^b^	3.69 ± 0.96^b^	3.94 ± 0.82^abc^	3.94 ± 0.68^b^	4.13 ± 1.13^b^
		**1.5%**	3.50 ± 1.28^b^	3.88 ± 0.69^b^	4.06 ± 0.94^abc^	3.75 ± 1.04^b^	4.13 ± 1.46^b^
	**Cranberry pomace**	**0.5%**	3.81 ± 0.65^b^	4.25 ± 0.83^b^	4.13 ± 0.92^abc^	3.94 ± 0.50^b^	4.13 ± 0.93^b^
		**1%**	3.94 ± 1.03^b^	4.06 ± 0.76^b^	4.25 ± 1.02^bc^	4.00 ± 1.07^b^	4.44 ± 1.16^b^
		**1.5%**	4.31 ± 1.20^b^	4.25 ± 0.89^b^	4.13 ± 1.28^abc^	4.25 ± 1.04^b^	4.50 ± 0.73^b^
	***Scutellaria baicalensis***	**0.5%**	4.19 ± 1.13^b^	3.94 ± 0.83^b^	3.88 ± 0.69^abc^	4.00 ± 0.92^b^	4.25 ± 0.52^b^
		**1%**	4.13 ± 1.16^b^	3.88 ± 1.41^b^	3.88 ± 0.98^abc^	4.19 ± 1.06^b^	4.63 ± 1.41^b^
		**1.5%**	4.25 ± 1.22^b^	4.00 ± 0.74^b^	3.56 ± 0.72^abc^	3.63 ± 0.88^b^	4.38 ± 0.89^b^

Results are expressed as mean ± standard deviation (SD). Results followed by the same lowercase letter(s) do not differ significantly (*p* > 0.05).

**Table 2:** Changes in textural profile by way of hardness, chewiness, gumminess, graininess, and greasiness across the various marinated oven-grilled chicken breast meat samples compared to the controls.

			Hardness	Chewiness	Gumminess	Graininess	Greasiness
**Control**	**Control**	**0%**	4.25 ± 1.39^a^	3.63 ± 1.19^ab^	2.88 ± 1.13^ab^	2.50 ± 0.92^ab^	2.25 ± 1.04^abcde^
	**Grape pomace**	**0.5%**	4.25 ± 1.75^a^	3.38 ± 1.30^ab^	2.88 ± 0.99^ab^	3.38 ± 1.13^ab^	2.25 ± 1.16^abcde^
		**1%**	3.13 ± 1.55^a^	2.63 ± 1.06^a^	2.13 ± 1.25^ab^	2.63 ± 1.41^ab^	2.00 ± 1.07^abcd^
		**1.5%**	4.13 ± 1.64^a^	3.25 ± 0.89^ab^	2.88 ± 1.25^ab^	3.25 ± 1.46^ab^	2.63 ± 1.85^abcde^
	**Cranberry pomace**	**0.5%**	3.13 ± 1.55^a^	2.88 ± 1.36^a^	3.00 ± 1.20^ab^	2.88 ± 1.49^ab^	4.63 ± 2.33^de^
		**1%**	3.75 ± 1.75^a^	3.75 ± 1.04^ab^	3.50 ± 1.41^ab^	2.88 ± 2.07^ab^	3.75 ± 2.49^bcde^
		**1.5%**	4.50 ± 2.45^a^	3.50 ± 1.07^ab^	3.25 ± 1.04^ab^	4.25 ± 1.36^b^	3.50 ± 1.85^abcde^
	***Scutellaria baicalensis***	**0.5%**	3.56 ± 1.58^a^	3.78 ± 1.60^ab^	3.78 ± 2.00^ab^	2.57 ± 1.41^ab^	1.56 ± 0.92^ab^
		**1%**	4.25 ± 1.91^a^	3.38 ± 1.69^ab^	4.00 ± 2.78^ab^	2.25 ± 1.06^ab^	2.50 ± 1.77^abcde^
		**1.5%**	3.38 ± 1.69^a^	3.75 ± 1.67^ab^	3.69 ± 1.71^ab^	3.00 ± 1.68^ab^	3.63 ± 3.02^abcde^
**African Spices**	**Control**	**0%**	2.88 ± 1.89^a^	2.38 ± 1.30^a^	2.00 ± 1.31^a^	2.63 ± 1.31^ab^	2.50 ± 1.41^abcde^
	**Grape pomace**	**0.5%**	4.25 ± 1.04^a^	2.88 ± 1.13^a^	2.56 ± 1.24^ab^	3.13 ± 1.60^ab^	2.25 ± 1.10^abcde^
		**1%**	3.00 ± 1.77^ab^	2.75 ± 1.67^a^	3.06 ± 1.43^ab^	2.50 ± 1.41^ab^	1.88 ± 0.83^abc^
		**1.5%**	5.00 ± 1.51^a^	3.88 ± 1.96^ab^	4.00 ± 2.67^ab^	2.88 ± 1.83^ab^	2.75 ± 1.91^abcde^
	**Cranberry pomace**	**0.5%**	3.25 ± 1.28^a^	3.50 ± 1.60^ab^	3.25 ± 1.83^ab^	2.75 ± 1.46^ab^	3.75 ± 2.49^bcde^
		**1%**	4.13 ± 1.96^a^	3.75 ± 1.49^ab^	4.00 ± 1.51^ab^	3.38 ± 1.25^ab^	3.38 ± 1.77^abcde^
		**1.5%**	3.63 ± 2.45^a^	4.13 ± 1.46^ab^	4.25 ± 1.49^b^	2.88 ± 1.49^ab^	4.00 ± 2.20^bcde^
	***Scutellaria baicalensis***	**0.5%**	4.14 ± 1.02^a^	4.21 ± 1.13^ab^	3.29 ± 0.65^ab^	2.56 ± 0.95^ab^	3.57 ± 0.53^abcde^
		**1%**	4.13 ± 0.92^a^	3.44 ± 0.83^ab^	2.88 ± 0.71^ab^	2.38 ± 1.07^ab^	2.38 ± 0.46^abcde^
		**1.5%**	4.00 ± 2.62^a^	3.88 ± 2.17^ab^	3.06 ± 1.37^ab^	2.44 ± 1.85^ab^	3.00 ± 2.14^abcde^
**Industrial pickle**	**Control**	**0%**	3.50 ± 1.31^a^	2.88 ± 0.83^a^	3.00 ± 1.20^ab^	2.63 ± 1.06^ab^	3.50 ± 1.07^abcd^
	**Grape pomace**	**0.5%**	3.75 ± 1.75^a^	3.75 ± 1.75^ab^	3.25 ± 1.28^ab^	2.88 ± 1.36^ab^	2.00 ± 1.07^abcd^
		**1%**	2.88 ± 1.13^a^	2.69 ± 0.96^a^	2.88 ± 1.13^ab^	2.75 ± 1.04^ab^	2.38 ± 1.30^abcde^
		**1.5%**	3.00 ± 1.69^a^	3.38 ± 1.85^ab^	2.50 ± 0.93^ab^	2.75 ± 1.67^ab^	2.38 ± 1.41^abcde^
	**Cranberry pomace**	**0.5%**	3.88 ± 1.36^a^	3.38 ± 1.69^ab^	3.38 ± 1.19^ab^	3.00 ± 1.31^ab^	4.25 ± 2.55^bcde^
		**1%**	2.88 ± 1.55^a^	2.63 ± 1.60^a^	2.81 ± 1.41^ab^	2.75 ± 1.49^ab^	4.88 ± 2.70^e^
		**1.5%**	3.25 ± 1.58^a^	3.25 ± 1.83^ab^	3.13 ± 1.55^ab^	2.50 ± 1.51^ab^	4.75 ± 2.49^e^
	***Scutellaria baicalensis***	**0.5%**	4.50 ± 2.00^a^	4.00 ± 1.41^ab^	3.88 ± 2.23^ab^	3.00 ± 2.00^ab^	3.00 ± 2.14^abcde^
		**1%**	3.88 ± 1.46^a^	3.63 ± 1.77^ab^	3.50 ± 1.41^ab^	2.50 ± 1.51^ab^	3.25 ± 2.19^abcde^
		**1.5%**	4.50 ± 2.20^a^	4.25 ± 2.12^ab^	3.63 ± 2.26^ab^	3.00 ± 2.33^ab^	3.63 ± 2.72^abcde^

Results are expressed as mean ± standard deviation (SD). Results followed by the same lowercase letter(s) do not differ significantly (*p* > 0.05).

**Table 3:** (a–c) Changes in L* a* b* color across the various marinated oven-grilled chicken breast meat samples incorporating (a) cranberry pomace (CP), (b) grape pomace (GP), and (c) Baikal skullcap (BS) compared to the control.

**(a) cranberry Pomace (CP) before and after Oven-Grill**						
	**CP before Oven-Grill**			**CP after Oven-Grill**		
**Samples**	L*	a*	b*	L*	a*	b*
1	54.2 ± 1.3^a^	0.5 ± 0.2^cd^	7.41 ± 1.1^bc^	76.4^a^ ± 1.8	−1.8 ± 0.1^c^	18.6 ± 2.1^de^
2	48.8 ± 3.2^bcd^	5.8 ± 1.3^a^	3.2 ± 0.5^cd^	67.7^bc^ ± 2.5	1.6 ± 1.2^b^	12.0 ± 2.5^g^
3	51.5 ± 2.6^abc^	2.1 ± 1.7^bcd^	5.4 ± 0.7^cd^	73.3^ab^ ± 4.5	−0.9 ± 3.4^bc^	16.7 ± 1.0^ef^
4	48.4 ± 1.4^bcd^	5.6 ± 1.1^a^	2.6 ± 1.5^d^	70.5^b^ ± 0.3	0.2 ± 0.4^abc^	14.0 ± 1.1^fg^
5	51.2 ± 5.3^abcd^	2.5 ± 0.6^bc^	12.9 ± 1.6^a^	68.2^bc^ ± 2.8	1.2 ± 1.5^ab^	20.7 ± 0.7^bcd^
6	46.1 ± 4.3^d^	3.6 ± 1.0^b^	11.9^ab^ ± 0.4	69.6^bc^ ± 4.2	0.2 ± 0.8^abc^	21.5 ± 1.0^bcd^
7	52.5 ± 2.2^ab^	3.6 ± 2.1^b^	14.3^a^ ± 5.9	68.5^bc^ ± 3.9	0.6 ± 0.8^abc^	20.2 ± 0.7^cd^
8	47.3 ± 0.5^bcd^	3.2 ± 0.8^b^	10.8^ab^ ± 2.8	64.3^cd^ ± 3.3	3.0 ± 1.3^a^	21.7 ± 2.3^bcd^
9	52.5 ± 2.0^ab^	0.3 ± 0.9^d^	14.8^a^ ± 1.5	71.3^cb^ ± 1.9	0.1 ± 1.4^abc^	28.2 ± 3.9^a^
10	47.1 ± 1.2^cd^	2.3 ± 1.1^cd^	10.3^ab^ ± 3.8	68.0^bc^ ± 5.5	0.8 ± 1.4^abc^	22.6 ± 1.5^bc^
11	49.7 ± 1.9^abcd^	3.4 ± 0.8^b^	14.6^a^ ± 2.4	60.2^d^ ± 2.4	2.8 ± 1.1^a^	24.0 ± 2.4^b^
12	50.8 ± 2.1^abcd^	2.5 ± 0.8^bc^	12.6^a^ ± 1.3	69.8^bc^ ± 0.3	0.4 ± 0.7^abc^	21.7 ± 1.1^bcd^
(b) grape pomace (GP) before and after oven-grill.						
	GP before oven-grill			GP after oven-grill		
Samples	L*	a*	b*	L*	a*	b*
1	52.1 ± 2.9^abc^	1.6 ± 0.9^cde^	4.9 ± 1.1^de^	83.3 ± 1.1^a^	0.8 ± 0.9^f^	15.7 ± 1.0^ef^
2	50.7 ± 1.8^abcd^	1.3 ± 0.8^de^	5.4 ± 3.9^de^	75.6 ± 3.0^bc^	1.5 ± 0.7^ef^	14.8 ± 1.4^f^
3	53.8 ± 3.6^a^	1.1 ± 0.5^e^	2.4 ± 1.3^e^	78.0 ± 3.4^ab^	1.7 ± 1.0^def^	12.4 ± 3.8^f^
4	49.4 ± 3.4^abcd^	2.0 ± 0.1^abcde^	6.1 ± 2.9^de^	69.8 ± 6.3^cde^	3.3 ± 0.7^abcd^	13.2 ± 1.9^f^
5	49.2 ± 0.9^abcd^	1.9 ± 0.5^bcde^	13.4 ± 1.6^b^	76.5 ± 3.5^abc^	2.4 ± 0.4^bcdef^	20.7 ± 2.1^cd^
6	43.7 ± 2.9^d^	3.5 ± 1.2^a^	13.3 ± 2.4^b^	73.0 ± 2.3^bcd^	2.2 ± 0.1^cdef^	19.9 ± 1.8^d^
7	46.1 ± 0.6^bcd^	3.5 ± 0.2^ab^	18.5 ± 0.8^a^	69.1 ± 5.4^cde^	3.8 ± 1.4^abc^	19.5 ± 2.3^de^
8	53.4 ± 6.5^ab^	3.1 ± 0.6^abc^	9.5 ± 2.2^bcd^	65.7 ± 2.7^de^	4.5 ± 1.2^a^	19.6 ± 1.8^de^
9	51.9 ± 4.4^abc^	2.4 ± 1.0^abcde^	12.6 ± 0.5^bc^	66.8 ± 5.2^de^	3.8 ± 1.0^abc^	29.1 ± 1.4^a^
10	46.5 ± 1.7^abcd^	2.8 ± 1.4^abcd^	13.4 ± 5.5^b^	66.0 ± 1.6^de^	4.1 ± 0.4^ab^	25.7 ± 2.6^ab^
11	52.1 ± 2.4^abc^	1.4 ± 0.6^de^	13.5 ± 3.3^b^	63.8 ± 4.2^e^	4.0 ± 0.1^ab^	26.0 ± 2.4^ab^
12	45.0 ± 2.6^cd^	2.3 ± 0.6^abcde^	8.1 ± 0.4^cd^	71.2 ± 5.0^bcde^	3.2 ± 1.4^abcde^	24.0 ± 2.3^bc^
(c) Baikal skullcap (BS) before and after oven-grill						
	BS before oven-grill			BS after oven-grill		
Samples	L*	a*	b*	L*	a*	b*
1	45.0 ± 0.1^bcd^	−0.3 ± 0.4^bcd^	4.3 ± 2.5^e^	77.0 ± 1.7^a^	−1.8 ± 0.4^cd^	18.8 ± 1.0^c^
2	48.7 ± 3.5^abc^	−0.9 ± 0.2^cde^	8.7 ± 0.6^cd^	73.6 ± 5.0^abc^	−2.6 ± 0.1^d^	17.3 ± 5.2^c^
3	44.8 ± 4.7^bcd^	−0.6 ± 0.3^cde^	7.0 ± 0.8^de^	74.2 ± 1.2^abc^	−2.5 ± 0.8^d^	15.8 ± 1.3^c^
4	46.2 ± 1.0^bcd^	−1.6 ± 1.0^e^	11.5 ± 3.8^bcd^	71.4 ± 2.5^abcd^	−2.2 ± 0.4^d^	18.0 ± 1.5^c^
5	49.2 ± 0.9^ab^	1.9 ± 0.5^a^	13.4 ± 1.6^ab^	76.5 ± 3.5^ab^	2.4 ± 0.4^ab^	20.7 ± 2.1^bc^
6	41.7 ± 2.4^d^	0.7 ± 1.1^b^	15.9 ± 3.3^ab^	63.0 ± 9.1^de^	1.6 ± 2.6^ab^	24.8 ± 4.8^ab^
7	44.0 ± 2.9^cd^	0.3 ± 0.3^bc^	14.6 ± 1.8^ab^	61.5 ± 2.1^e^	1.5 ± 1.0^b^	25.3 ± 0.6^ab^
8	42.6 ± 1.5^d^	0.3 ± 0.1^bc^	17.2 ± 2.7^a^	61.4 ± 9.2^e^	0.3 ± 2.6^bc^	25.4 ± 2.4^ab^
9	51.9 ± 4.4^ab^	2.4 ± 1.0^a^	12.6 ± 0.5^abc^	66.8 ± 5.2^bcde^	3.8 ± 1.0^a^	29.1 ± 1.4^a^
10	44.3 ± 3.4^bcd^	−0.2 ± 0.1^bcd^	15.0 ± 3.8^ab^	68.3 ± 6.7^abcde^	−1.1 ± 0.3^cd^	26.9 ± 1.6^a^
11	48.9 ± 0.8^abc^	−1.7 ± 0.6^e^	14.4 ± 2.3^ab^	64.8 ± 2.2^cde^	−1.4 ± 1.0^cd^	25.5 ± 2.3^ab^
12	45.6 ± 0.5^bcd^	−1.1 ± 0.5^de^	14.7 ± 2.0^ab^	63.5 ± 4.7^de^	0.4 ± 0.1^bc^	28.6 ± 3.1^a^

Key: Results are expressed as mean ± standard deviation (SD) at the probability level of *p* < 0.05. The numbers representing the samples are as follows: (1) control (antioxidant additive % = 0.0); (2) control (antioxidant additive % = 0.5); (3) control (antioxidant additive % = 1.0); (4) control (antioxidant additive % = 1.5); (5) AS (antioxidant additive % = 0.0); (6) AS (antioxidant additive % = 0.5); (7) AS (antioxidant additive % = 1.0); (8) AS (antioxidant additive % = 1.5); (9) IM (antioxidant additive % = 0.0); (10) IM (antioxidant additive % = 0.5); (11) IM (antioxidant additive % = 1.0); (12) IM (antioxidant additive % = 1.5). African spice: AS; and industrial marinade/pickle: IM.

**Table 4:** Changes in textural cutting force across the various marinated oven-grilled chicken breast meat samples compared to control.

Antioxidant Additive	Marinade Type	Percentage (%) of Antioxidant Additive	Chicken Cutting Force [N]
Cranberry pomace	Control	0.0	17.3 ± 1.0^cdefghi^
		0.5	13.4 ± 2.8^abcde^
		1.0	17.1 ± 2.5^cdefghi^
		1.5	15.8 ± 3.6^abcdefgh^
	AS	0.0	12.8 ± 1.5^abcd^
		0.5	15.2 ± 6.5^abcdefgh^
		1.0	19.2 ± 7.9^cdefghi^
		1.5	18.2 ± 0.8^cdefghi^
	IM	0.0	21.4 ± 2.7^hi^
		0.5	19.4 ± 6.1^defghi^
		1.0	17.7 ± 2.3^cdefghi^
		1.5	19.4 ± 2.2^efghi^
Grape Pomace	Control	0.0	19.1 ± 4.0^cdefghi^
		0.5	20.2 ± 4.3^fghi^
		1.0	18.6 ± 1.0^cdefghi^
		1.5	20.3 ± 3.0^ghi^
	AS	0.0	19.2 ± 3.4^cdefghi^
		0.5	16.9 ± 2.0^bcdefgh^
		1.0	14.0 ± 2.5^abcdefg^
		1.5	15.4 ± 1.6^abcdefgh^
	IM	0.0	16.0 ± 2.1^abcdefgh^
		0.5	13.7 ± 2.1^abcdef^
		1.0	14.9 ± 3.6^abcdefgh^
		1.5	14.9 ± 2.2^abcdefgh^
BS	Control	0.0	12.6 ± 1.0^abc^
		0.5	21.0 ± 2.5^hi^
		1.0	10.5 ± 1.0^ab^
		1.5	19.2 ± 1.2^defghi^
	AS	0.0	12.8 ± 1.5^abcd^
		0.5	15.7 ± 1.5^abcdefgh^
		1.0	10.2 ± 1.5^a^
		1.5	19.1 ± 4.4^cdefghi^
	IM	0.0	16.0 ± 2.1^abcdefgh^
		0.5	23.5 ± 4.8^i^
		1.0	18.4 ± 3.1^cdefghi^
		1.5	19.1 ± 3.6^cdefghi^

Key: Results are expressed as mean ± standard deviation (SD). Results followed by same lowercase letter(s) in the column of cutting force do not differ significantly (*p* > 0.05); African spice: AS; industrial marinade/pickle: IM; and Baikal skullcap: BS.

## Data Availability

Data is contained within the article.

## References

[1] Sas A. (2022). Production of Meat and Meat Products in Poland 2018–2021, by Product Type, Agriculture-Farming, Statistica. https://www.statista.com/statistics/1125806/poland-production-of-meat-and-meat-products/.

[2] Adamski M., Kuzniacka J., Milczewska N. (2017). Preferences of consumers for choosing poultry meat. Pol. J. Natur. Sci..

[3] Sosnówka-Czajka E., Skomorucha I., Muchacka R. (2017). Effect of Organic Production System on the Performance and Meat Quality of Two Purebred Slow-Growing Chicken Breeds. Ann. Anim. Sci..

[4] Pawłowska J., Sosnówka-Czajka E. (2019). Factors affecting chick quality in Poland. World’s Poult. Sci. J..

[5] Kokoszyński D., Żochowska-Kujawska J., Kotowicz M., Sobczak M., Piwczyński D., Stęczny K., Majrowska M., Saleh M. (2022). Carcass characteristics and selected meat quality traits from commercial broiler chickens of different origin. Anim. Sci. J..

[6] Jaturasitha S., Kayan A., Wicke M. (2008). Carcass and meat characteristics of male chickens between Thai indigenous compared with improved layer breeds and their crossbred. Arch. Anim. Breed..

[7] De Liu X., Jayasena D.D., Jung Y., Jung S., Kang B.S., Heo K.N., Lee J.H., Jo C. (2012). Differential Proteome Analysis of Breast and Thigh Muscles between Korean Native Chickens and Commercial Broilers. Asian-Australas. J. Anim. Sci..

[8] Swatland H. (2008). How pH causes paleness or darkness in chicken breast meat. Meat Sci..

[9] Martini S., Cattivelli A., Conte A., Tagliazucchi D. (2021). Black, green, and pink pepper affect differently lipid oxidation during cooking and in vitro digestion of meat. Food Chem..

[10] Zhang H., Wu J., Guo X. (2016). Effects of antimicrobial and antioxidant activities of spice extracts on raw chicken meat quality. Food Sci. Hum. Wellness.

[11] Istrati D., Ciuciu A.M., Vizireanu C., Ionescu A., Carballo J. (2015). Impact of Spices and Wine-Based Marinades on Tenderness, Fragmentation of Myofibrillar Proteins and Color Stability in Bovine B iceps Femoris Muscle. J. Texture Stud..

[12] Sokołowicz Z., Augustyńska-Prejsnar A., Krawczyk J., Kačániová M., Kluz M., Hanus P., Topczewska J. (2021). Technological and Sensory Quality and Microbiological Safety of RIR Chicken Breast Meat Marinated with Fermented Milk Products. Animals.

[13] Cheok C., Chin N., Yusof Y., Kamal S.M.M., Sazili A. (2011). Effect of marinating temperatures on physical changes of traditionally marinated beef satay. J. Food Process. Preserv..

[14] Lemos A., Nunes D., Viana A. (1999). Optimization of the still-marinating process of chicken parts. Meat Sci..

[15] Al-Dalali S., Li C., Xu B. (2021). Evaluation of the effect of marination in different seasoning recipes on the flavor profile of roasted beef meat via chemical and sensory analysis. J. Food Biochem..

[16] Salmon C., Knize M., Felton J. (1997). Effects of marinating on heterocyclic amine carcinogen formation in grilled chicken. Food Chem. Toxicol..

[17] Shahidi F., Hossain A. (2018). Bioactives in spices, and spice oleoresins: Phytochemicals and their beneficial effects in food preservation and health promotion. J. Food Bioact..

[18] Jalali M., Mahmoodi M., Moosavian S.P., Jalali R., Ferns G., Mosallanezhad A., Imanieh M.H., Mosallanezhad Z. (2020). The effects of ginger supplementation on markers of inflammatory and oxidative stress: A systematic review and meta-analysis of clinical trials. Phytother. Res..

[19] Roopchand D.E., Krueger C.G., Moskal K., Fridlender B., Lila M.A., Raskin I. (2013). Food-compatible method for the efficient extraction and stabilization of cranberry pomace polyphenols. Food Chem..

[20] Teplá J.L., Dostálová T., Lužová D., Rožnovská J., Přichystalová L., Kalhotka L., Dvořák L., Sýkora V., Šustová K. (2013). Antimicrobial effects of selected plant extracts on the shelf life of goat whey. MendelNet.

[21] Yu J., Ahmedna M., Goktepe I. (2005). Effects of processing methods and extraction solvents on concentration and antioxidant activity of peanut skin phenolics. Food Chem..

[22] Zhang Y., Henning S.M., Lee R.-P., Huang J., Zerlin A., Li Z., Heber D. (2015). Turmeric and black pepper spices decrease lipid peroxidation in meat patties during cooking. Int. J. Food Sci. Nutr..

[23] Awuchi C.G., Okpala C.O.R. (2022). Natural nutraceuticals, especially functional foods, their ma-jor bioactive components, formulation, and health benefits for disease prevention-An overview. J. Food Bioact..

[24] Raut S.S., Rindhe S.N., Verma S.K., Swami J.N., Mundhe B.L., Rayeesul I. (2015). Effect of Acidulant on Chicken Pickle Incorporated with Poultry By-products. J. Meat Sci. Technol..

[25] Viegas O., Amaro L.F., Ferreira I.M., Pinho O. (2012). Inhibitory Effect of Antioxidant-Rich Marinades on the Formation of Heterocyclic Aromatic Amines in Pan-Fried Beef. J. Agric. Food Chem..

[26] Richardson P. (2004). Improving the Thermal Processing of Foods.

[27] Schröder M.J. (2003). Food Quality and Consumer Value: Delivering Food that Satisfies.

[28] Ježek F., Kameník J., Macharáčková B., Bogdanovičová K., Bednář J. (2020). Cooking of meat: Effect on texture, cooking loss and microbiological quality—A review. Acta Vet. Brno.

[29] Beckett F. (2012). Sausage & Mash.

[30] Liao G., Wang G., Xu X., Zhou G. (2010). Effect of cooking methods on the formation of heterocyclic aromatic amines in chicken and duck breast. Meat Sci..

[31] Farhadian A., Jinap S., Abas F., Sakar Z.I. (2010). Determination of polycyclic aromatic hydrocarbons in grilled meat. Food Control.

[32] Kerth C.R., Blair-Kerth L.K., Jones W.R. (2003). Warner-Bratzler shear force repeatability in beef longissimus steaks cooked with a convection oven, broiler, or clam-shell grill. J. Food Sci..

[33] Khan M.I., Min J.-S., Lee S.-O., Yim D.G., Seol K.-H., Lee M., Jo C. (2015). Cooking, storage, and reheating effect on the formation of cholesterol oxidation products in processed meat products. Lipids Health Dis..

[34] Bai W.K., Zhang F.J., He T.J., Su P.W., Ying X.Z., Zhang L.L., Wang T. (2016). Dietary pro-biotic Bacillus subtilis strain fmbj increases antioxidant capacity and oxidative stability of chicken breast meat during storage. PLoS ONE.

[35] Lengkidworraphiphat P., Wongpoomchai R., Taya S., Jaturasitha S. (2020). Effect of genotypes on macronutrients and antioxidant capacity of chicken breast meat. Asian-Australas. J. Anim. Sci..

[36] Augustyńska-Prejsnar A., Ormian M., Sokołowicz Z. (2018). Physicochemical and Sensory Properties of Broiler Chicken Breast Meat Stored Frozen and Thawed Using Various Methods. J. Food Qual..

[37] Brambila G.S., Bowker B.C., Zhuang H. (2016). Comparison of sensory texture attributes of broiler breast fillets with different degrees of white striping. Poult. Sci..

[38] Akmakçı S., Topdaş E.F., Kalın P., Han H., Şekerci P., Köse L.P., Gülçin İ. (2015). Antioxidant capacity and functionality of oleaster (Elaeagnus angustifolia L.) flour and crust in a new kind of fruity ice cream. Int. J. Food Sci. Technol..

[39] Civille G.V., Carr B.T. (2015). Sensory Evaluation Techniques.

[40] Barido F.H., Lee S.K. (2022). Effect of detoxified Rhus verniciflua extract on oxidative stability and quality improvement of raw chicken breast during cold storage. J. Anim. Sci. Technol..

[41] Luciano G., Moloney A.P., Priolo A., Röhrle F.T., Vasta V., Biondi L., López-Andrés P., Grasso S., Monahan F.J. (2011). Vitamin E and polyunsaturated fatty acids in bovine muscle and the oxidative stability of beef from cattle receiving grass or concentrate-based rations1. J. Anim. Sci..

[42] Ali S., Kang G.-H., Yang H.-S., Jeong J.-Y., Hwang Y.-H., Park G.-B., Joo S.-T. (2007). A Comparison of Meat Characteristics between Duck and Chicken Breast. Asian-Australas. J. Anim. Sci..

[43] Kopec W., Jamroz D., Wiliczkiewicz A., Biazik E., Pudlo A., Korzeniowska M., Hikawczuk T., Skiba T. (2020). Antioxidative Characteristics of Chicken Breast Meat and Blood after Diet Supplementation with Carnosine, L-histidine, and β-alanine. Antioxidants.

[44] Augustyńska-Prejsnar A., Ormian M., Sokołowicz Z. (2017). The influence of frozen storage duration and thawing methods on the meat quality of broiler chickens. Apar. Badaw. i Dydakt..

[45] Floegel A., Kim D.O., Chung S.J., Song W.O., Fernandez M.L., Bruno R.S., Koo S.I., Chun O.K. (2010). Development and validation of an algorithm to establish a total antioxidant capacity database of the US diet. Int. J. Food Sci. Nutr..

[46] Sáyago-Ayerdi S., Brenes A., Viveros A., Goñi I. (2009). Antioxidative effect of dietary grape pomace concentrate on lipid oxidation of chilled and long-term frozen stored chicken patties. Meat Sci..

[47] Kim J., Bang J., Beuchat L.R., Kim H., Ryu J.-H. (2012). Controlled fermentation of kimchi using naturally occurring antimicrobial agents. Food Microbiol..

[48] Lee K.J., Jung P.M., Oh Y.-C., Song N.-Y., Kim T., Ma J.Y. (2014). Extraction and Bioactivity Analysis of Major Flavones Compounds fromScutellaria baicalensisUsing In Vitro Assay and Online Screening HPLC-ABTS System. J. Anal. Methods Chem..

[49] Smaoui S., Ben Hlima H., Ghorbel R. (2012). The effect of sodium lactate and lactic acid combinations on the microbial, sensory, and chemical attributes of marinated chicken thigh. Poult. Sci..

[50] Warner R., Miller R., Ha M., Wheeler T.L., Dunshea F., Li X., Wheeler T. (2021). Meat tenderness: Underlying mechanisms, instrumental measurement, and sensory assessment. Meat Muscle Biol..

[51] Chumngoen W., Tan F.J. (2015). Relationships between descriptive sensory attributes and physico-chemical analysis of broiler and Taiwan native chicken breast meat. Asian-Australas. J. Anim. Sci..

[52] Schilling M.W., Schilling J.K., Claus J.R., Marriott N.G., Duncan S.E., Wang H. (2003). Instrumental texture assessment and consumer acceptability of cooked broiler breasts evaluated using a geometrically uniform-shaped sample. J. Muscle Foods.

[53] Chuaynukool K., Wattanachant S., Siripongvutikorn S. (2007). Chemical and physical properties of raw and cooked spent hen, broiler and Thai indigenous chicken muscles in mixed herbs acidified soup (Tom Yum). J. Food Technol..

[54] Amaral A.B., da Silva M.V., Lannes S.C.D.S. (2018). Lipid oxidation in meat: Mechanisms and protective factors—A review. Food Sci. Technol..

[55] Sáyago-Ayerdi S., Brenes A., Goñi I. (2009). Effect of grape antioxidant dietary fiber on the lipid oxidation of raw and cooked chicken hamburgers. LWT-Food Sci. Technol..

[56] Murphy R.Y., Marks B.P. (2000). Effect of meat temperature on proteins, texture, and cook loss for ground chicken breast patties. Poult. Sci..

[57] Rabeler F., Feyissa A.H. (2018). Kinetic Modeling of Texture and Color Changes During Thermal Treatment of Chicken Breast Meat. Food Bioprocess Technol..

